# Aberrant lncRNA–mRNA expression profile and function networks during the adipogenesis of mesenchymal stem cells from patients with ankylosing spondylitis

**DOI:** 10.3389/fgene.2022.991875

**Published:** 2022-10-03

**Authors:** Shuizhong Cen, Mingxi Cai, Yihan Wang, Xiuyi Lu, Zhipeng Chen, Haobo Chen, Yingdong Fang, Changping Wu, Sujun Qiu, Zhenhua Liu

**Affiliations:** ^1^ Department of Spine Surgery, Zhujiang Hospital, Southern Medical University, Guangzhou, China; ^2^ Department of Dermatology, The Fourth Affiliated Hospital of Guangzhou Medical University, Guangzhou, China; ^3^ Department of Orthopedics, Sun Yat-sen Memorial Hospital, Sun Yat-sen University, Guangzhou, China; ^4^ Department of Orthopedics, People’s Hospital of Taishan, Jiangmen, China

**Keywords:** ankylosing spondylitis, mesenchymal stem cells, adipogenic differentiation, lncRNA, mRNA

## Abstract

**Objective:** We have already demonstrated that mesenchymal stem cells from patients with ankylosing spondylitis (ASMSCs) exhibited greater adipogenic differentiation potential than those from healthy donors (HDMSCs). Here, we further investigated the expression profile of long noncoding RNA (lncRNA) and mRNA, aiming to explore the underlying mechanism of abnormal adipogenic differentiation in ASMSCs.

**Methods:** HDMSCs and ASMSCs were separately isolated and induced with adipogenic differentiation medium for 10 days. Thereafter, lncRNAs and mRNAs that were differentially expressed (DE) between HDMSCs and ASMSCs were identified *via* high-throughput sequencing and confirmed by quantitative real-time PCR (qRT–PCR) assays. Then, the DE genes were annotated and enriched by GO analysis. In addition, protein interaction network was constructed to evaluate the interactions between DE mRNAs and to find hub nodes and study cliques. Besides, co-expression network analysis was carried out to assess the co-expressions between DE mRNA and DE lncRNAs, and competing endogenous RNA (ceRNA) network analysis were conducted to predict the relationships among lncRNAs, mRNAs and miRNAs. The signaling pathways based on the DE genes and the predicted DE genes were enriched by KEGG analysis.

**Results:** A total of 263 DE lncRNAs and 1376 DE mRNAs were found during adipogenesis in ASMSCs. qRT–PCR indicated that the expression of the top 20 mRNAs and the top 10 lncRNAs was consistent with the high-throughput sequencing data. Several lncRNAs (NR_125386.1, NR_046473.1 and NR_038937.1) and their target genes (SPN and OR1AIP2), together with the significantly co-expressed pairs of DE lncRNAs and DE mRNAs (SLC38A5-ENST00000429588.1, TMEM61-ENST00000400755.3 and C5orf46-ENST00000512300.1), were closely related to the enhanced adipogenesis of ASMSCs by modulating the PPAR signaling pathway.

**Conclusion:** Our study analyzed the expression profiles of DE lncRNAs and DE mRNAs during adipogenesis in ASMSCs and HDMSCs. Several DE lncRNAs, DE mRNAs and signaling pathways that probably participate in the aberrant adipogenesis of ASMSCs were selected for future study. These results will likely provide potential targets for our intervention on fat metaplasia and subsequent new bone formation in patients with AS in the future.

## Introduction

Ankylosing spondylitis (AS) is a common inflammatory arthritis characterized by chronic inflammation and pathological new bone formation (Navarro-Compan et al., 2021). With the use of anti-tumor necrosis factor-α agents, nonsteroidal anti-inflammatory drugs and disease-modifying anti-rheumatic drugs, low back pain and peripheral joint pain caused by chronic inflammation can be significantly relieved in most patients (Ward et al., 2019). However, structural damage and disability, resulting from pathological new bone formation, seem inevitable for them (Ritchlin and Adamopoulos, 2021). Thus, further study the pathogenesis of new bone formation and provide new treatment options are urgently needed.

Fat metaplasia, with enhanced signals on T1-weighted sequences and reduced signals on short tau inversion recovery sequences, is an important magnetic resonance imaging (MRI) finding in patients with AS (Maksymowych et al., 2014). It can be frequently observed in the sacroiliac joint or the vertebral corner, where inflammation and new bone formation mainly occur (Machado et al., 2016). Recently, evidence has shown that this MRI signal corresponds to a high level of adipocyte accumulation (Baraliakos et al., 2019), which is a vital intermediary step and a strong contributor to pathological new bone formation in AS (Chiowchanwisawakit et al., 2011). Therefore, elucidating the cause of fat metaplasia is important.

Mesenchymal stem cells (MSCs) are a heterogeneous population of cells with fibroblast-like morphology and immunomodulation potentials, as well as self-renewal and multipotent differentiation capacities (Uccelli et al., 2008). As an origin of both fat and bone, MSCs obviously contribute to the abnormal adipocyte accumulation and thus the pathologic structural changes in AS. Our previous study demonstrated that MSCs from patients with AS (ASMSCs) had a significantly enhanced adipogenic capacity compared with those from healthy donors (HDMSCs), potentially leading to adipocyte accumulation and fat metaplasia (Liu et al., 2019). However, the specific mechanism of abnormal ASMSC adipogenesis remains undefined.

Long noncoding RNAs (lncRNAs) are RNA transcripts containing more than 200 nucleotides but without protein-coding potential (Ransohoff et al., 2018). Through interacting with DNA, RNAs and/or proteins, they play crucial roles in various cellular processes, including cell differentiation, the cell cycle and metabolism (Bridges et al., 2021). Evidence is accumulating that lncRNAs not only participate in the adipogenic differentiation of MSCs, but also contribute to the pathogenesis of AS (Sun et al., 2022). Based on these findings, it is reasonable for us to presume that the enhanced adipogenesis of ASMSCs may be related to abnormal lncRNA expression.

In this study, we compared the lncRNA and mRNA expression profiles of both ASMSCs and HDMSCs and analyzed the possible mechanism underlying the expression difference, aiming to reveal the role lncRNAs may play in the abnormal adipogenesis of ASMSCs and provide insights into the pathogenesis of new bone formation.

## Materials and methods

### Ethics and enrollment

Our study was approved by the Ethics Committee of Zhujiang Hospital, Southern Medical University, Guangzhou, China. After obtaining written informed consent, a punch in the posterior upper iliac crest was generated after local anesthesia with 2% lidocaine, and bone marrow samples were extracted from 12 healthy donors and 10 patients with AS (diagnosed according to the New York Revised Criteria (van der Linden et al., 1984)). Detailed characteristics of these volunteers are presented in Supplementary Table S1 **.**

### Cell isolation and culture

HDMSCs and ASMSCs were separately purified from the bone marrow samples of healthy donors and AS patients using density gradient centrifugation as described (Colter et al., 2000). To be specific, 20 ml bone marrow sample was diluted 1:1 with Hanks’ balanced salt solution (HBSS, GIBCO, United States) and layered over about 10 ml of Ficoll (Ficoll-Paque, Pharmacia, Sweden). After centrifugation (2,500 × g, 30 min), the mononuclear cell layer was extracted from the interface, suspended in HBSS, then centrifuged (1,500 × g, 15 min) and resuspended in Dulbecco’s modified Eagle’s medium (DMEM, Gibco, United States) supplemented with 10% heat-inactivated fetal bovine serum (FBS, Gibco, United States). Afterwards, they were seeded in 25-cm^2^ flasks and cultured in an incubator with 5% CO_2_ at 37°C. To remove the suspended cells, the medium was replaced 48 h later and every 3 days thereafter. At 80–90% confluence, the adherent cells were harvested using 0.25% Trypsin containing 0.53 mM EDTA (Gibco, United States) and re-plated in 75-cm^2^ flasks as passage 1 of MSCs. MSCs were expanded and used for adipogenic differentiation at passages 4. Flow cytometry was performed to identify the surface markers of MSCs by detecting the expression of CD29-PE, CD14-APC, CD44-FITC, CD105-FITC, CD45-APC and HLA DR-PerCP (all the antibodies were purchased from BD, United States).

### Adipogenic differentiation induction

HDMSCs and ASMSCs were seeded in 12-well plates at a density of 5 × 10^4^ cells/well in growth medium containing high-glucose DMEM supplemented with 10% FBS. When the cells reached 80–90% confluence, the medium was replaced with adipogenic differentiation medium consisting of high-glucose DMEM supplemented with 10% FBS, 0.2 mM indomethacin (Sigma–Aldrich, Germany), 10 μg/ml insulin (Sigma–Aldrich, Germany), 0.5 mM 3-isobutyl-1-methylxanthine (Sigma–Aldrich, Germany) and 1 μM dexamethasone (Sigma–Aldrich, Germany). The adipogenic differentiation medium was replaced every 3 days. HDMSCs and ASMSCs on the 10th day of induction were harvested for the following experiments.

### Oil red O staining and Triglyceride quantification

As described in our previous study (Liu et al., 2019; Cen et al., 2020), cells were firstly washed with phosphate-buffered saline and then fixed with 4% formaldehyde for 20 min. Thereafter, formaldehyde was removed, and the cells were washed with 60% isopropanol and then stained with 0.2% ORO (Sigma-Aldrich, Germany) for 30 min. Afterwards, cells were washed with phosphate-buffered saline and observed under a microscope (Olympus, Japan). Stained oil droplets were dissolved in 100% isopropanol and quantified by detecting the optical absorbance (500 nm) with a spectrophotometer (Thermo Fisher, Germany). Triglyceride content was quantified using a triglyceride quantification kit (ab178780, Abcam, United Kingdom), according to manufacturer’s instructions.

### Western blot analysis

Proteins were extracted from HDMSCs and ASMSCs on the 10th day of induction, and then quantified as previously described (Liu et al., 2019). Equal amounts of protein extracts were denatured, separated and then transferred onto polyvinylidene fluoride membranes (Millipore, United States). Afterwards, membranes were blocked with 5% skim milk and incubated overnight at 4°C with primary antibodies against GAPDH, peroxisome proliferator-activated receptor gamma (PPAR-γ), fatty acid binding protein 4 (FABP4), adiponectin (all from Abcam, United Kingdom). Horseradish peroxidase -conjugated immunoglobulin IgG (Santa Cruz, United States) was used as secondary antibody and incubated with the membranes at room temperature. The immunoreactive bands were then visualized using the Immobilon Western Chemiluminescent HRP Substrate (Millipore, United States).

### Library construction and sequencing

On the 10th day of induction, total RNAs were respectively extracted from HDMSCs and ASMSCs using the TRIzol (Invitrogen, United States) according to the manufacturer’s instructions, and ribosomal RNA was removed using the Ribo-Zero™ kit (Epicentre, Madison, WI, United States). Fragmented RNA (the average length was approximately 200 bp) were subjected to first strand and second strand cDNA synthesis following by adaptor ligation and enrichment with a low-cycle according to instructions of NEBNext^®^ Ultra™ RNA Library Prep Kit for Illumina (NEB, United States). The purified library products were evaluated using the Agilent 2200 TapeStation and Qubit^®^2.0(Life Technologies, United States). The libraries were paired-end sequenced (PE150, Sequencing reads were 150 bp) at Guangzhou RiboBio Co., Ltd. (Guangzhou, China) using Illumina-HiSeq 3000 platform.

### Quality control and expression analysis

In order to remove the low-quality data, the Raw fastq sequences were treated with Trimmomatic tools (v 0.36) using the following options: TRAILING:20, SLIDINGWINDOW:4:15 MINLEN:52, to remove trailing sequences below a phred quality score of 20 and to achieve uniform sequence lengths for downstream clustering processes. Thereafter, the relative high-quality data were normalized to the expected number of reads per kilobase of transcript per million mapped reads (RPKM). Differentially expressed (DE) genes were detected by the DESeq2 method based on negative binomial generalized linear models. Genes with significant fold changes (log2-fold change >1; Q-value < 0.05) between HDMSCs and ASMSCs were accepted for further study.

### Quantitative real-time polymerase chain reaction

To confirm the reliability of RNA sequencing, qRT–PCR was performed as previously described (Liu et al., 2017). Simply put, total RNAs were extracted from HDMSCs and ASMSCs on the 10th day of induction, and then transcribed into complementary DNA by PrimeScript RT reagent kit (Takara, Japan). qRT–PCR was conducted with a Light Cycler^®^ 480 PCR System (Roche, Switzerland). Data were normalized to GAPDH, and the relative expression level of each gene was analyzed using the 2^−ΔCt^ method.

### GO and KEGG pathway analysis

The annotation and enrichment information of DE genes were analyzed by Gene Ontology (GO) term enrichment analysis using KOBAS3.0 software (http://www.geneontology.org). Label classification for gene function and gene product attributes, including cellular component (CC), molecular function (MF) and biological process (BP), were detailed. The biological pathways of the DE genes were categorized by KEGG pathway analysis using KOBAS 3.0 software (http://www.genome.jp/kegg). After being calculated using Fisher’s exact test, signal transduction and disease pathway enrichment of DE genes with *p* values <0.05 were mapped using KEGG pathway annotation.

### Interaction analysis and co-expression network construction

To obtain information on all target-related genes or proteins, PPI network was constructed by using the STRING database and visualized in Cytoscape software as described (confidence score was set as score >0.4) (Doncheva et al., 2019). GENEMANIA (http://genemania.org/search/) was used to evaluate the interactions between them (Warde-Farley et al., 2010). Additionally, the MCODE app was used to find hub nodes and study cliques in the PPI network (degree cutoff = 2, max. Depth = 100, k-core = 2, and node score cutoff = 0.2) (Bader and Hogue, 2003). After obtaining the overall interaction relationship, the Pearson correlation coefficients and *p* values between multiple genes were calculated. lncRNAs and mRNAs with COR ≤0.85 and *p* value ≥ 0.05 were excluded, and the mRNA–lncRNA co-expression network was also built by Cytoscape software (https://cytoscape.org).

### Competing endogenous RNA network analysis

The above-selected mRNAs and lncRNAs for co-expression network analysis were also used to predict miRNA in the miRbase. Then, miRNAs obtained from miRbase were further screened using the miRanda and TargetScan programs. Afterward, RNA22 was used to predict those lncRNAs and mRNAs with miRNA recognition elements (MREs) for the targeted miRNAs. Finally, we constructed the competitive ceRNA network using Cytoscape software.

### Statistical analysis

Quantitative data are expressed as the means ± standard deviations (SD). Spearman correlation was used to assess the relationship between lncRNAs and their target genes. Student’s t test was performed to analyze the statistical significance between two groups. The abovementioned statistical analysis was performed using SPSS 23.0 software (Chicago), and *p* values < 0.05 indicated statistical significant.

## Results

### ASMSCs had greater adipogenic capacity than HDMSCs

**FIGURE 1:**
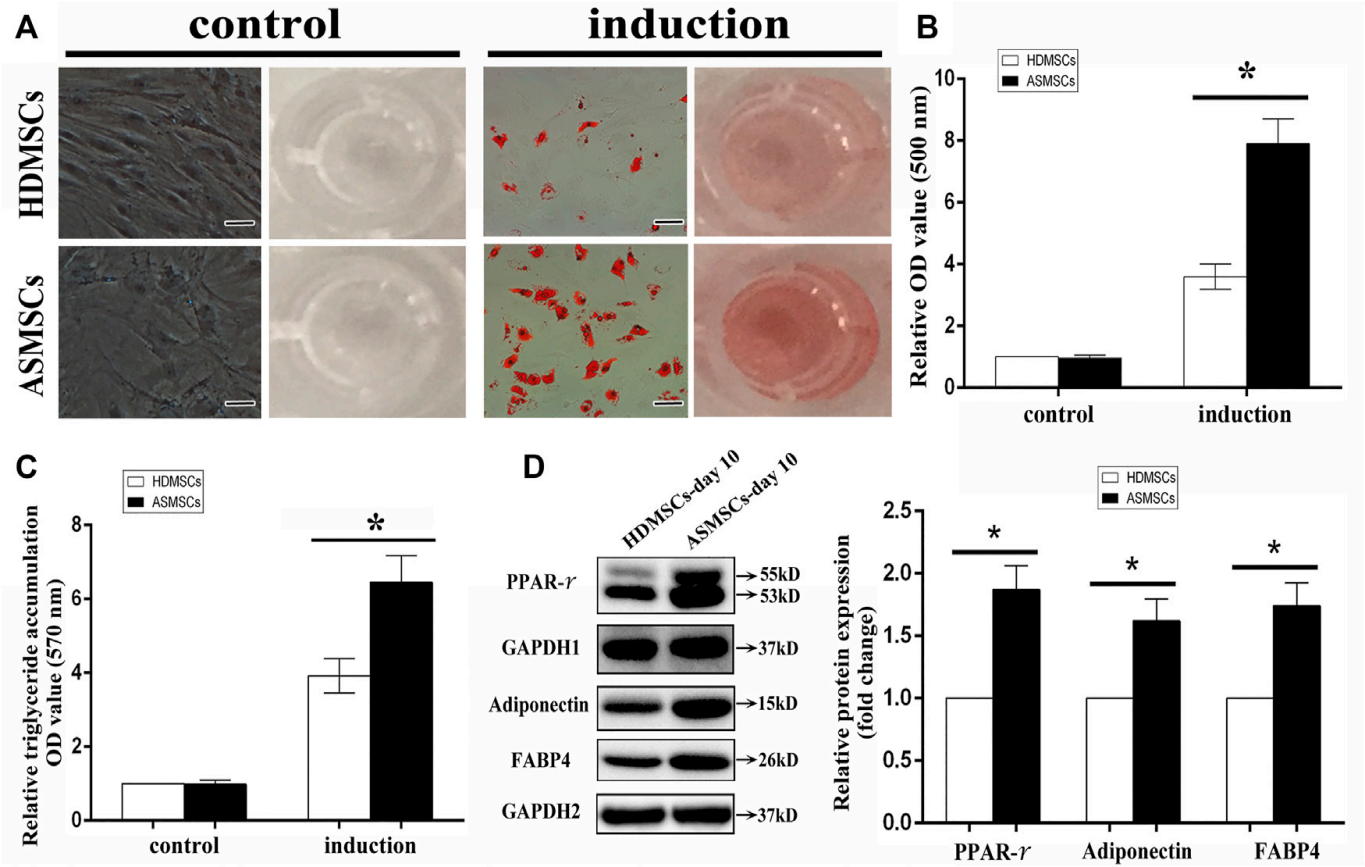
ASMSCs had greater adipogenic capacity than HDMSCs. The adipogenic differentiation potential of HDMSCs and ASMSCs were assessed *via* ORO staining and quantification and further confirmed by quantification of triglyceride accumulation. **(A)** ORO staining indicated that ASMSCs displayed more intense staining on Day 10 (100X, scale bar indicate 100 um). Consistent results were also observed in quantifying of ORO staining **(B)** and triglyceride accumulation **(C)**. Moreover, protein expressions of PPAR-γ, FABP4 and adiponectin were higher in ASMSCs than those of HDMSCs **(D)**. *p < 0.05.

Both HDMSCs and ASMSCs used in our study were plastic-adherent cells with spindle-shaped, and they were negative for CD14, CD45 and HLA-DR and positive for CD29, CD44 and CD105 (Supplementary Figure S1), which conformed to the criteria stated by the International Society for Cellular Therapy (Dominici et al., 2006). HDMSCs and ASMSCs were cultured in adipogenic medium for 0–10 days. Cells were stained with ORO on Days 0 and 10, and the stained oil droplets were dissolved in 100% isopropanol. ASMSCs exhibited more intense staining on Day 10 than HDMSCs (Figure 1A). Consistent results were also observed in the quantification of ORO staining (Figure 1B) and triglyceride accumulation (Figure 1C). Moreover, protein expressions of the adipogenic differentiation markers (PPAR-γ, FABP4, adiponectin) were higher in ASMSCs than those of HDMSCs (Figure 1D). As with our previous report (Liu et al., 2019), these data supported the notion that ASMSCs had greater adipogenic capacity than HDMSCs.

### Expression profile of DE mRNAs and DE lncRNAs during adipogenesis

**FIGURE 2:**
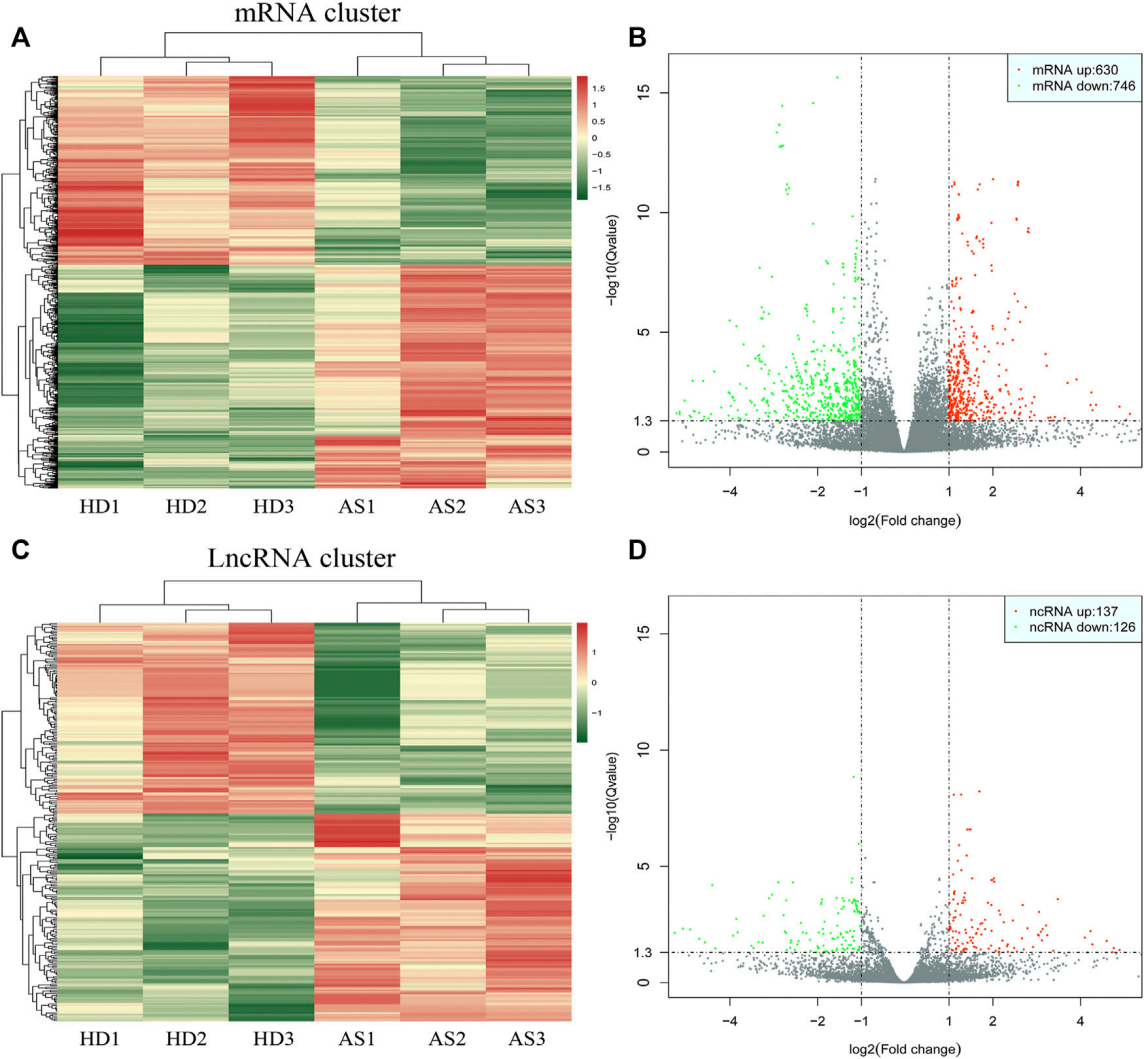
Expression profile of DE mRNAs and DE lncRNAs during adipogenesis. **(A)** Heatmaps of DE mRNAs between HDMSCs and ASMSCs. **(B)** Volcano plots of DE mRNAs between HDMSCs and ASMSCs. A total of 630 upregulated mRNAs and 746 downregulated mRNAs were found. **(C)** Heatmaps of DE lncRNAs between HDMSCs and ASMSCs. **(D)** Volcano plots of DE lncRNAs between HDMSCs and ASMSCs. A total of 137 upregulated mRNAs and 126 downregulated lncRNAs were found. In the heatmap, HD indicates MSCs from healthy donors, and AS indicates MSCs from patients with ankylosing spondylitis.

As depicted in the clustergram (Figure 2A) and volcano plot (Figure 2B), a total of 1376 DE mRNAs were found between HDMSCs and ASMSCs. Among them, 630 mRNAs were upregulated, while 746 mRNAs were downregulated in ASMSCs. As previously described (Li M. et al., 2019), the top 20 DE mRNAs with largest fold change were chosen for further analysis and listed in Table 1. Adipogenesis-related mRNAs, including GJB2, ADRA1A, SYT1, LPO, BICDL2, TBATA, APELA, CCKAR, RAPGEF3, NTSR1 and CDH10, may be related to abnormal adipogenesis of ASMSCs. As shown in the clustergram (Figure 2C) and volcano plot (Figure 2D), 137 upregulated and 126 downregulated lncRNAs were also found in ASMSCs. As previously described (Wang et al., 2021), the top 10 DE lncRNAs with largest fold change were chosen for further analysis and shown in Table 2. These data revealed a significant genetic difference between HDMSCs and ASMSCs during adipogenesis.

**TABLE 1:** The characteristics of the top 20 mRNA with largest fold change.

Gene name	Accession no.	Fold change	Regulation
GJB2	NM_004004.5	−8.030699999	Down
KLHL4	NM_057162.2	−6.893522024	Down
ADRA1A	NM_001322502.1	6.838047793	Up
SYT1	NM_001135805.1	−6.353658659	Down
LPO	NM_001160102.1	−6.079573971	Down
BICDL2	NM_001103175.1	−6.013239434	Down
TBATA	NM_001318243.1	5.992884615	Up
APELA	NM_001297550.1	−5.971761787	Down
CCDC144NL	NM_001004306.2	5.739184535	Up
GJA5	NM_181703.3	−5.722018948	Down
KNDC1	NM_001347865.1	−5.668406921	Down
NXNL1	NM_138454.1	5.6332394	Up
CFAP47	NM_001304548.1	5.56203244	Up
CCKAR	NM_000730.2	−5.552178045	Down
RAPGEF3	NM_001098532.2	−5.52055224	Down
CDH7	NM_004361.3	−5.4848221	Down
NTSR1	NM_002531.2	−5.484635344	Down
ACP7	NM_001004318.2	−5.481850292	Down
CDH10	NM_001317222.1	5.154448318	Up
RNF43	NM_001305545.1	−5.120399674	Down

**TABLE 2:** The characteristics of the top 10 LncRNA with largest fold change.

Accession no.	Fold change	Regulation	Chromosome	Strand	Start	End	Class	Size (bp)
ENST00000546836.1	7.089031716	Up	12	+	70913986	70932443	antisense	551
NR_023925.1	6.794368371	Up	18	-	1268311	1359629	intergenic	2231
ENST00000593060.1	−6.32530761	Down	19	+	55006193	55048086	antisense	786
ENST00000592405.1	−6.277156326	Down	18	-	55108311	55119598	intergenic	576
ENST00000429588.1	−6.249747719	Down	21	+	36430360	36481070	antisense	893
ENST00000432265.1	5.870811734	Up	7	+	95596682	95613719	intergenic	771
NR_051996.1	5.806070453	Up	5	-	122436497	122479087	antisense	1424
ENST00000400755.3	−5.41952407	Down	1	+	142618771	142679074	intergenic	601
ENST00000512300.1	−5.232310472	Down	5	-	147886086	147886878	intronic	793
NR_003948.2	−5.046339245	Down	6	+	31053450	31059890	intergenic	2183

### Confirmation of DE mRNAs and lncRNAs by qRT–PCR

**FIGURE 3:**
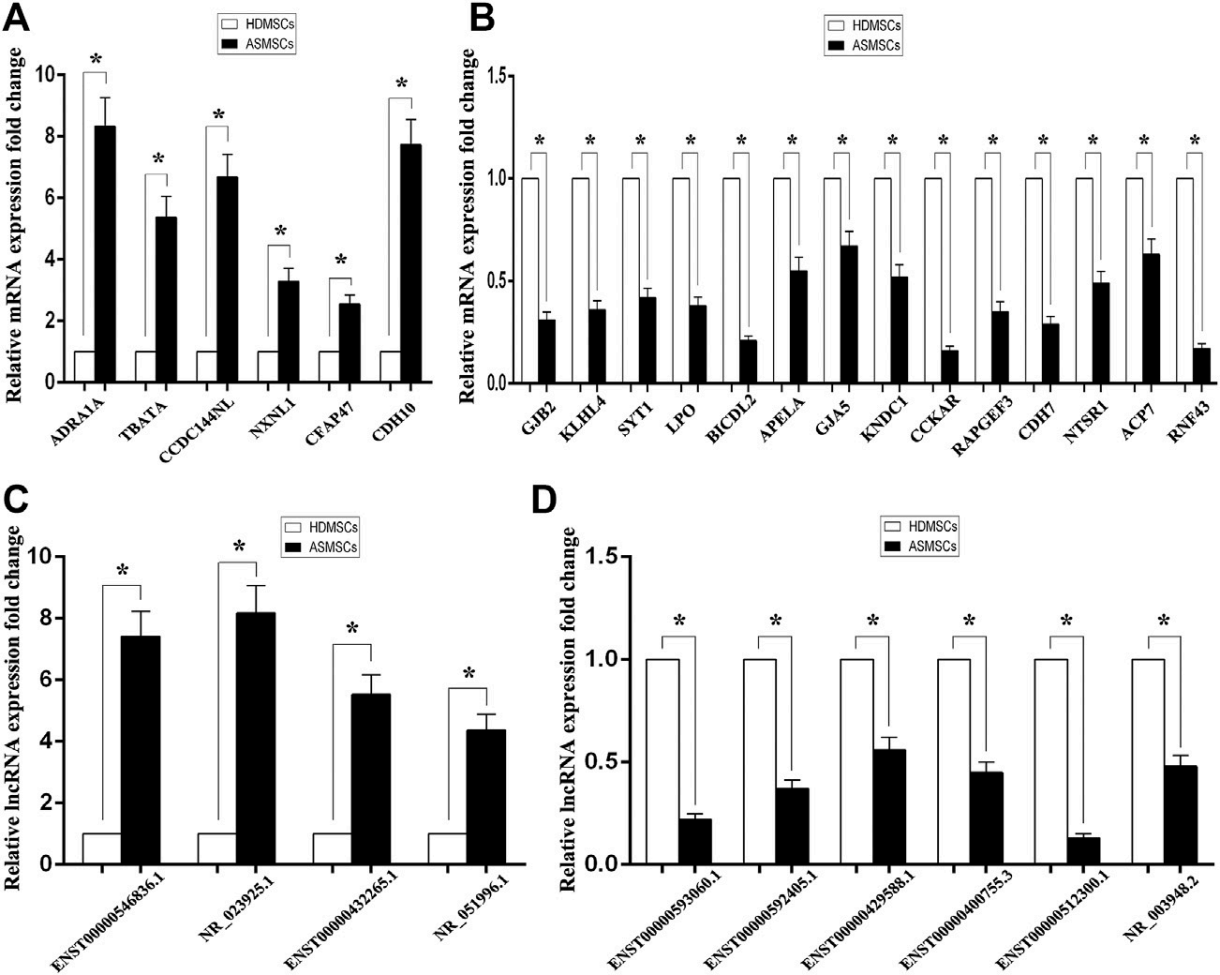
Confirmation of DE mRNAs and DE lncRNAs by qRT–PCR. The expression levels of the top 20 DE mRNAs and top 10 DE lncRNAs were confirmed *via* qRT–PCR. As shown in **(A)** and **(B)**, 6 mRNAs were upregulated and 14 mRNAs were downregulated in ASMSCs. As depicted in **(C)** and **(D)**, 4 lncRNAs were upregulated and 6 lncRNAs were downregulated in ASMSCs. Data are presented as the means ± SDs. *p < 0.05.

To detect the reliability of the RNA sequencing, the expression levels of the top 20 DE mRNAs and the top 10 DE lncRNAs were detected by qRT–PCR. Compared with HDMSCs, ADRA1A, TBATA, CCDC144NL, NXNL1, CFAP47 and CDH10 were upregulated in ASMSCs (Figure 3A), while GJB2, KLHL4, SYT1, etc., were downregulated in ASMSCs (Figure 3B). With regard to lncRNAs, the expression levels of ENST100000546836.1, NR_023925.1, ENST00000432265.1 and NR_051996.1 were increased in ASMSCs (Figure 3C), while the expression levels of ENST00000593060.1, ENST00000592405.1, ENST00000429588.1, ENST00000400755.3, ENST00000512300.1 and NR_003948.2 were decreased in ASMSCs (Figure 3D). These results were essentially comparable to the data shown in Table 1 and Table 2, which strongly confirmed the reliability and accuracy of the RNA sequence data.

### GO and KEGG pathway analysis

**FIGURE 4:**
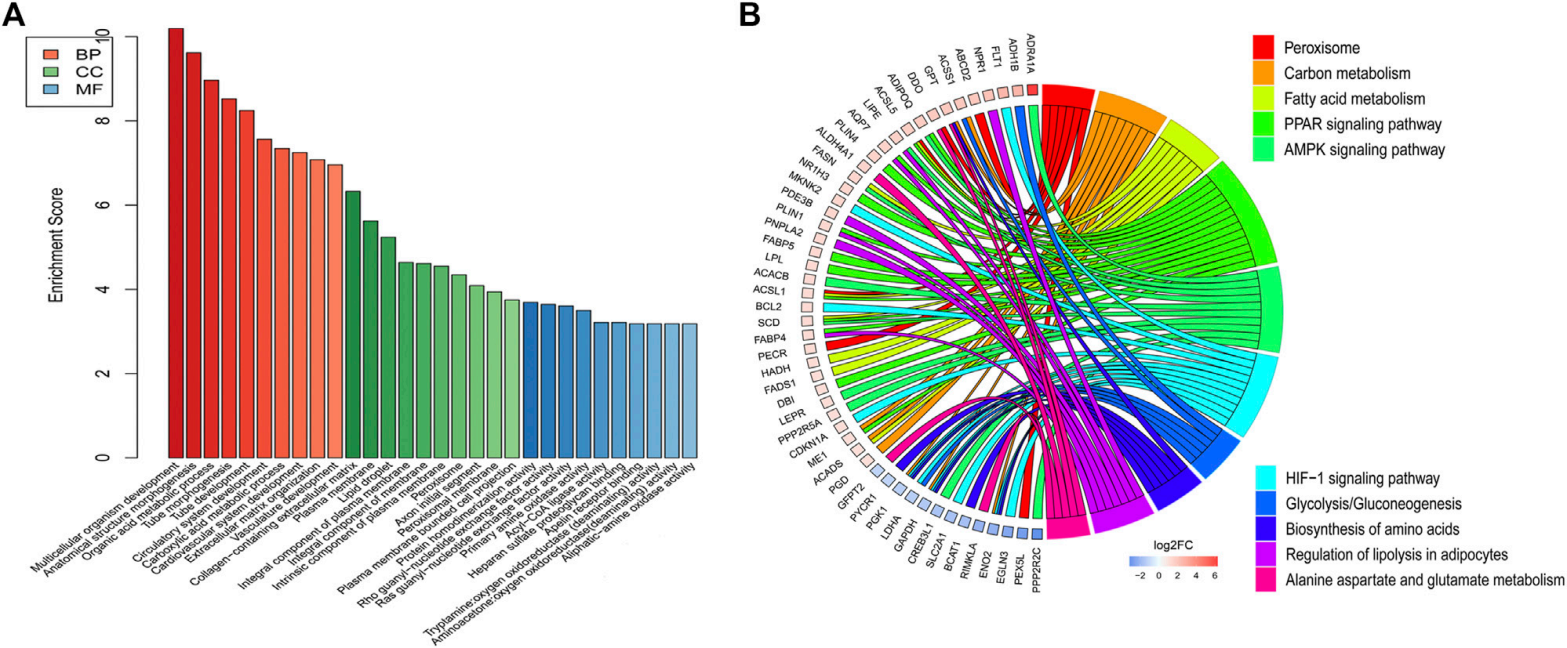
GO and KEGG pathway analysis. **(A)** Top 10 GO terms with the largest significant difference in the three domains. **(B)** Top 10 KEGG signaling pathways with the largest significant difference.

DE mRNAs between HDMSCs and ASMSCs were annotated and enriched by GO analysis. The top 10 GO terms of the three domains, including biological processes (BP), cellular components (CC) and molecular function (MF), are presented in Figure 4A and Table 3. Specifically, in the BP domain, the significantly enriched GO terms included multicellular organism development, anatomical structure morphogenesis, organic acid metabolic process, etc. In the CC domain, the significantly enriched GO terms were collagen-containing extracellular matrix, plasma membrane, lipid droplet, etc. In the MF domain, the significantly enriched GO terms were protein homodimerization activity, rho guanyl-nucleotide exchange factor activity, ras guanyl-nucleotide exchange factor activity, etc.

**TABLE 3:** Go analysis of the top 10 mRNA expression with largest significant difference in three domains.

Term	Domain	Count	*p*-value	FDR
Multicellular organism development	Biological Process	183	6.38908E−11	2.53519E−07
Anatomical structure morphogenesis	Biological Process	98	2.40776E−10	4.77699E−07
Organic acid metabolic process	Biological Process	57	1.08071E−09	1.42941E−06
Tube morphogenesis	Biological Process	43	2.99955E−09	2.97556E−06
Tube development	Biological Process	50	5.68753E−09	4.51363E−06
Circulatory system development	Biological Process	49	2.72992E−08	1.80539E−05
Carboxylic acid metabolic process	Biological Process	49	4.52575E−08	2.56546E−05
Cardiovascular system development	Biological Process	35	5.65196E−08	2.80337E−05
Extracellular matrix organization	Biological Process	27	8.34836E−08	3.6807E−05
Vasculature development	Biological Process	34	1.10453E−07	4.38276E−05
collagen-containing extracellular matrix	Cellular Component	29	4.72E−07	0.000196215
Plasma membrane	Cellular Component	189	2.38E−06	0.000495853
Lipid droplet	Cellular Component	11	5.8E−06	0.000803622
Integral component of plasma membrane	Cellular Component	69	2.3E−05	0.001952998
Integral component of membrane	Cellular Component	175	2.43E−05	0.001952998
Intrinsic component of plasma membrane	Cellular Component	71	2.82E−05	0.001952998
peroxisome	Cellular Component	13	4.53E−05	0.00268971
Axon initial segment	Cellular Component	5	8.19E−05	0.004256294
Peroxisomal membrane	Cellular Component	8	0.000115	0.005304842
Plasma membrane bounded cell projection	Cellular Component	79	0.000179	0.007427337
Protein homodimerization activity	Molecular Function	40	0.000203	0.041938016
Rho guanyl-nucleotide exchange factor activity	Molecular Function	9	0.000227	0.041938016
Ras guanyl-nucleotide exchange factor activity	Molecular Function	12	0.000248	0.041938016
Primary amine oxidase activity	Molecular Function	3	0.000318	0.041938016
acyl-CoA ligase activity	Molecular Function	4	0.000612	0.041938016
Heparan sulfate proteoglycan binding	Molecular Function	4	0.000612	0.041938016
Apelin receptor binding	Molecular Function	2	0.000659	0.041938016
Tryptamine: oxygen oxidoreductase (deaminating) activity	Molecular Function	2	0.000659	0.041938016
Aminoacetone: oxygen oxidoreductase (deaminating) activity	Molecular Function	2	0.000659	0.041938016
Aliphatic-amine oxidase activity	Molecular Function	2	0.000659	0.041938016

KEGG pathway analysis was performed to enrich the critical signaling pathways involved in the abnormal adipogenesis of ASMSCs. A total of 381 signaling pathways were enriched, and the top 10 pathways with the largest significant difference are shown in Figure 4B and Table 4. They are fatty acid metabolism, amino acid biosynthesis, carbon metabolism, peroxisome, alanine aspartate and glutamate metabolism, glycolysis/gluconeogenesis, regulation of lipolysis in adipocytes, PPAR signaling pathway, AMPK signaling pathway, and HIF-1 signaling pathway. Among them, the PPAR signaling pathway and related mRNAs might play important roles in the increased adipogenesis of ASMSCs.

**TABLE 4:** The top 10 pathways with largest significant difference in KEGG analysis.

Pathway	Count	*p*-Value	FDR	Gene
PPAR signaling pathway	13	1.03797E-07	2.77138E-05	ACSL1, ACSL5, ADIPOQ, AQP7, DBI, FABP4, FABP5, LPL, ME1, NR1H3, PLIN1, PLIN4, SCD
Fatty acid metabolism	8	0.00016654	0.017264094	ACADS, ACSL1, ACSL5, FADS1, FASN, HACD4, HADH, SCD
Biosynthesis of amino acids	9	0.000223653	0.017264094	ACO2, ACY1, BCAT1, ENO2, GAPDH, GPT, PGK1, PYCR1, TPI1
Carbon metabolism	11	0.000378972	0.017264094	ACADS, ACO2, ACSS1, CAT, ENO2, GAPDH, GPT, ME1, PGD, PGK1, TPI1
Peroxisome	9	0.000440632	0.017264094	ABCD2, ACSL1, ACSL5, CAT, DDO, ECH1, PECR, PEX11A, PEX5L
Alanine aspartate and glutamate metabolism	6	0.000501051	0.017264094	ALDH4A1, DDO, GFPT2, GPT, NAT8L, RIMKLA
AMPK signaling pathway	11	0.000507481	0.017264094	ACACB, ADIPOQ, ADRA1A, CREB3L1, FASN, LEPR, LIPE, PPARGC1A, PPP2R2C, PPP2R5A, SCD
Glycolysis/Gluconeogenesis	8	0.000517276	0.017264094	ACSS1, ADH1B, ALDH3A2, ENO2, GAPDH, LDHA, PGK1, TPI1
Regulation of lipolysis in adipocytes	7	0.000785134	0.022982087	AQP7, FABP4, LIPE, NPR1, PDE3B, PLIN1, PNPLA2
HIF-1 signaling pathway	10	0.000902812	0.022982087	BCL2, CDKN1A, EGLN3, ENO2, FLT1, GAPDH, LDHA, MKNK2, PGK1, SLC2A1

### Interaction and co-expression network analysis

**FIGURE 5:**
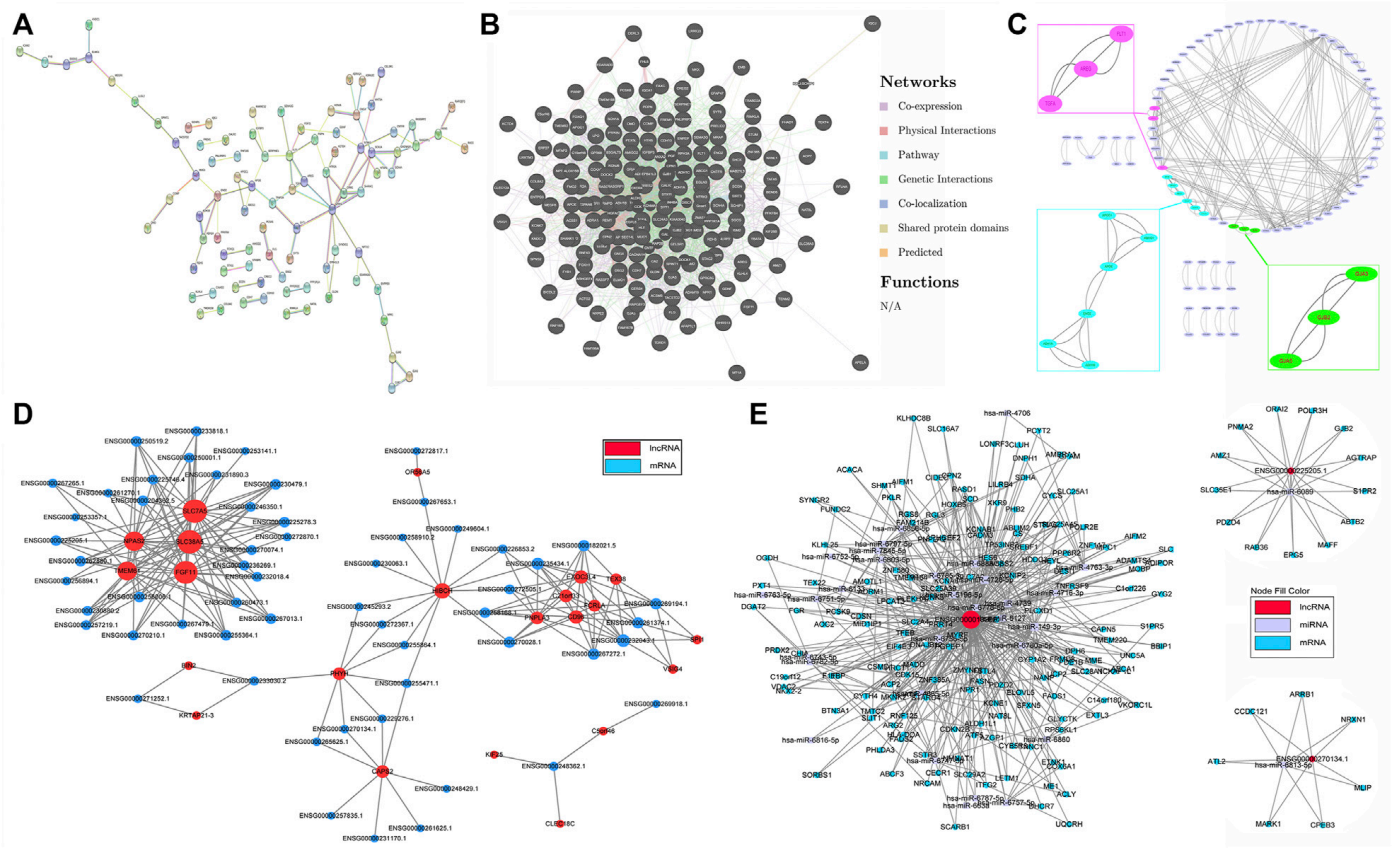
Interaction and co-expression network analysis. **(A)** Protein–protein interaction (PPI) network based on DE mRNAs. **(B)** The specific interactions between DE genes in the PPI network. **(C)** The significant hub nodes and molecular complex clustered by the MCODE in the PPI network. **(D)** Co-expression network of DE lncRNAs and DE mRNAs. **(E)** ceRNA network of DE lncRNAs, mRNAs and their predicted miRNAs.

Illustrating the molecular interaction helps to clarify the pathophysiology and reveal the underlying mechanisms of disease. Proteins encoded by DE mRNAs were analyzed using PPI network. Our data suggested that GRIA1, FLT1, APOE, ENO2, GNG4, NTRK3 and SCN1A interacted with much more DE genes than others, implying that they might be important targets contributing to the abnormal adipogenesis of AS (Figure 5A and Supplementary Table S2). Meanwhile, the specific interactions between DE genes were shown in Figure 5B and Supplementary Table S3. A total of 2349 interactions between DE genes were identified. Among them, 1275 were co-expression, 732 were genetic interaction, 111 were physical interaction, 106 were shared protein domains, 89 were co-localization, 34 were pathway-related and 2 were predicted. Besides, module analysis was also performed to find and study cliques in the PPI network. As shown in Figure 5C and Supplementary Table S4, the top 3 clusters and their hub nodes were: GJA3, GJB2 and GJA5 cluster, AREG, TGFA and FLT1 cluster, ENO2, ADH1A, APOC1, ADH1B, APOE and ABCG1 cluster.

Thereafter, interactions between DE lncRNAs and DE mRNAs were analyzed, and the top 30 pairs are shown in Figure 5D after filtering the repetitions. Within the network, SLC7A5, SLC38A, FGF11, TMEM61, NPAS2, HIBCH, c21orf33, PNPLA3, FCRLA and their co-expressed lncRNAs were significantly enriched. The top 10 co-expression pairs are listed in Table 5. ceRNA network analysis is necessary to reveal the interactions among lncRNAs, mRNAs and miRNAs. As shown in Figure 5E, 32 predicted miRNAs were significantly enriched and combined with 3 lncRNAs and 182 mRNAs. Among them, miR-6778-5p, miR-6127, miR-6089, miR-6813-5p and miR-149-3p interacted with much more mRNAs than others, indicating that they might play critical roles in the mechanism of enhanced adipogenesis in ASMSCs.

**TABLE 5:** The top 10 co-expression pairs.

mRNA	Gene	LncRNA	Gene	Correlation coefficient	*p*-value
NM_206966.2	C5orf46	ENST00000512300.1	ENSG00000248362.1	0.999622608	2.48E-05
NM_001145474.3	TEX38	ENST00000567002.1	ENSG00000261374.1	0.998940695	0.002976
NM_004112.3	FGF11	ENST00000575310.1	ENSG00000262880.1	0.998449462	2.48E-05
NM_001077594.1	EXOC3L4	ENST00000426350.1	ENSG00000182021.5	0.997913396	0.017857
NM_001318889.1	CD96	ENST00000426350.1	ENSG00000182021.5	0.997913396	0.017857
NM_182532.2	TMEM61	ENST00000400755.3	ENSG00000230880.2	0.997399331	0.017857
NM_033518.3	SLC38A5	ENST00000429588.1	ENSG00000230479.1	0.997324702	7.44E-04
NM_002518.3	NPAS2	ENST00000414948.1	ENSG00000236269.1	0.996942475	2.48E-05
NM_198047.2	HIBCH	ENST00000513071.1	ENSG00000245293.2	0.996585754	2.48E-05
NM_001184867.1	FCRLA	ENST00000598595.1	ENSG00000182021.5	0.996532408	7.19E-04

### Target prediction of lncRNAs

**FIGURE 6:**
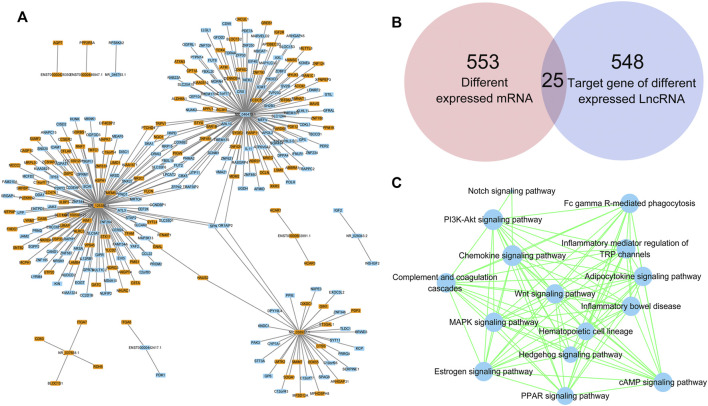
Target prediction of lncRNAs. **(A)** DE lncRNAs and their target genes. Blue indicates downregulated, while orange indicates upregulated. **(B)** Intersection between DE mRNAs and target genes of DE lncRNAs. **(C)** KEGG pathway analysis based on the DE lncRNA-targeted genes.

lncRNAs regulate gene expression by interacting with proteins, RNAs and DNAs. The possible target genes of the DE lncRNAs were analyzed in this study, and the predictive genes with binding scores greater than 0.9 are shown in Figure 6A. NR_125386.1, NR_046473.1 and NR_038937.1 were the top 3 lncRNAs that appeared to have various target genes. SPN and OR1AIP2 were target genes of all the top 3 lncRNAs. HAUS2, TRPV1, PTCHD4, SKA1, NQO1, H6PD, ORAI2, RRP15, COX6B2, SLC35F6, FBXL18, PIGW, LPCAT2, FUT2, PNMA2, ZFP42, CBX5, UTP11 and TRAF3IP2 were target genes of two lncRNAs. In addition, the Venn diagram analysis showed that 25 genes existed both in 553 DE mRNAs and in 548 DE lncRNA target genes (Figure 6B). Moreover, the target genes of DE lncRNAs were clustered by KEGG pathway analysis (Figure 6C). The PPAR signaling pathway, adipocytokine signaling pathway, Wnt signaling pathway and the other 12 signaling pathways related to DE lncRNAs might play critical roles in the abnormal adipogenesis of ASMSCs.

## Discussion

The increased adipogenesis of ASMSCs may result in fat metaplasia and contribute to new bone formation. Further investigation of the underlying mechanism of increased adipogenesis of ASMSCs may help to elucidate the pathogenesis of AS. In this study, we performed high-throughput sequencing to analyze lncRNA and mRNA expression in ASMSCs and HDMSCs during adipogenesis. Our data demonstrated that a total of 263 lncRNAs and 1376 mRNAs were abnormally expressed, and the PPAR signaling pathway and its related mRNAs might be involved in the increased adipogenesis of ASMSCs. Further bioinformatics analysis indicated that several DE lncRNAs (ENST00000429588.1, ENST00000400755.3 & ENST00000512300.1) may be upstream targets contributing to abnormal ASMSC adipogenesis by acting as ceRNAs or by interacting with mRNAs.

MSCs are recognized as a promising and revolutionary treatment due to their multiple differentiation potential and immunomodulatory properties. An increasing number of studies have reported that MSCs can effectively alleviate inflammation and other symptoms of rheumatism (Shi et al., 2018). However, the abnormal behavior of MSCs may also result in autoimmune diseases, including AS (Krajewska-Wlodarczyk et al., 2017). Ye et al. reported that oxidative stress-mediated mitochondrial dysfunction facilitates senescence of ASMSCs and contributes to the pathogenesis of AS (Ye et al., 2020). Xie et al. demonstrated that TNF-α-mediated m^6^A modification of ELMO1 triggers directional migration of ASMSCs, which might lead to chronic inflammation of AS (Xie et al., 2021). Similarly, our previous data has shown that ASMSCs have enhanced adipogenic capacity (Liu et al., 2019). Given that an imbalance between adipogenesis and osteogenesis may lead to abnormal bone metabolism (Cen et al., 2020), our previous study may partly explain the phenomenon of fat metaplasia and new bone formation in patients with AS. Therefore, further study of the underlying mechanism of abnormal adipogenesis in ASMSCs is of significance.

lncRNAs have aroused great interest in medical and scientific circles due to their multifaceted and versatile regulatory roles in various biological processes. Numerous lncRNAs are reported to modulate the adipogenic differentiation process (Squillaro et al., 2020). For example, SRA, lnc-ORA and lncRNA-Adi can regulate the early stage of adipogenesis, while ADNCR, lncRNA Plnc1, lncRNA PVT1 and NEAT1 were suggested to target C/EBPα and/or PPARγ in the late stage of adipogenesis (Rey et al., 2021). In addition, lncRNAs have also been reported to be involved in the pathogenesis of AS and serve as biomarkers and/or potential therapeutic targets (Chen et al., 2019). For example, lncRNA-AK001085 and TUG1 can serve as diagnostic biomarkers for AS. LINC00311, NKILA and Lnc-ITSN1-2 can be used to monitor the activity and assess the prognosis of AS. H19 and MEG3 are potential therapeutic targets for AS (Sun et al., 2022). In our study, 137 upregulated and 126 downregulated lncRNAs were found in ASMSCs during adipogenesis as compared to HDMSCs. Among them, three of the top 10 DE lncRNAs (ENST00000429588.1, ENST00000400755.3 & ENST00000512300.1) appeared to be significantly co-expressed with DE mRNAs, implying that they may be important targets of abnormal adipogenesis. However, their specific role still needs to be further confirmed.

Considering that lncRNAs play regulatory roles in gene expression by interacting with various molecular species, bioinformatic analysis was performed to illuminate their interactions and provide directions for our future study. The PPI network analysis indicated that, in addition to our previously confirmed genes (PPARG, CEBPA and ADIPOQ) (Liu et al., 2019), other DE genes (GRIA1, FLT1, APOE, ENO2, GNG4, NTRK3 and SCN1A) warrant further study as well. Among them, it is worth noting that APOE was also a hub node gene in the modules analysis. Given the fact that APOE is a key player in adipogenesis (Chiba et al., 2003), it is reasonable to presume that APOE is involved in the abnormal adipogenesis of ASMSCs. Additionally, the co-expression analysis provided three promising co-expression candidates (SLC38A5-ENST00000429588.1, TMEM61-ENST00000400755.3 and C5orf46-ENST00000512300.1) involved in abnormal adipogenesis. Moreover, the ceRNA network analysis suggested that miR-6778-5p, miR-6127, miR-6089, miR-6813-5p and miR-149-3p were significantly enriched. Notably, miR-149-3p can regulate both the adipogenic and osteogenic differentiation of MSCs(Li Y. et al., 2019), while miR-6127 and miR-6089 are related to immunoregulation and inflammation (Tubita et al., 2019; Yang et al., 2020). Finally, target prediction analysis revealed that the lncRNAs (NR_125386.1, NR_046473.1 and NR_038937.1) and the target genes they shared (SPN and OR1AIP2) may play critical roles in the abnormal adipogenesis of ASMSCs through the PPAR signaling pathway.

The PPAR signaling pathway is one of the most important transcriptional modulators involved in both the early and late stages of adipogenic differentiation (Lee et al., 2019). In this study, the PPAR signaling pathway and its related mRNAs also drew our attention. KEGG analysis based on both DE mRNA- and DE lncRNA-targeted genes demonstrated that the PPAR signaling pathway was aberrantly activated and significantly enriched during adipogenesis in ASMSCs. Another study also found that serum from patients with AS could increase the expression of PPAR, the key molecule of the PPAR signaling pathway (Hu et al., 2015). Based on the recognized role of PPAR in adipogenic differentiation (Lefterova et al., 2014), we believe that the PPAR signaling pathway is an important cause and key target of fat metaplasia in AS. In addition, fatty acid metabolism (Xu et al., 2015), the HIF-1 signaling pathway (Ding et al., 2018) and alanine aspartate and glutamate metabolism (Zhou et al., 2020) were also reported to be related to AS, but further studies are still needed to explore whether they mediate abnormal adipogenesis.

In this study, we again confirmed the enhanced adipogenesis of ASMSCs. During adipogenesis, hundreds of DE lncRNAs and DE mRNAs were found in ASMSCs, and the potential regulatory mechanisms were explored by bioinformatics analysis. Several lncRNAs (NR_125386.1, NR_046473.1 and NR_038937.1) and their target genes (SPN and OR1AIP2), together with several co-expression pairs of DE lncRNAs and DE mRNAs (SLC38A5-ENST00000429588.1, TMEM61-ENST00000400755.3 and C5orf46-ENST00000512300.1), may play a central role in regulating the adipogenic differentiation of ASMSCs by activating the PPAR signaling pathway. These results could help to elucidate the pathogenesis of fat metaplasia and new bone formation in patients with AS and provide potential therapeutic targets for them. However, there are still some limitations in this study. For example, we did not clarify the specific function and relative mechanism of DE lncRNAs during adipogenesis. These limitations should be explored in depth in future studies.

## Data Availability

The datasets presented in this study can be found in online repositories. The names of the repository/repositories and accession number(s) can be found below: Gene Expression Omnibus accession number GSE212613. https://www.ncbi.nlm.nih.gov/geo/query/acc.cgi?acc=GSE212613

## Supplementary material

Supplementary Figure S1 HDMSCs and ASMSCs exhibited similar morphologies and phenotypes. **(A)** Both HDMSCs and ASMSCs were plastic-adherent cells with spindle-shaped. **(B)** HDMSCs and ASMSCs were both negative for CD14, CD45 and HLA-DR and positive for CD29, CD44 and CD105, indicating a typical MSC phenotype.
