# Supplementary material for: Aberrant lncRNA–mRNA expression profile and function networks during the adipogenesis of mesenchymal stem cells from patients with ankylosing spondylitis
Source: Front Genet. 2022 Oct 3;13:991875. doi: 10.3389/fgene.2022.991875 (PMC9563993; doi:10.3389/fgene.2022.991875)
Supplement: Supplementary file 1 [file Table1.docx]

**Supplemental Table 1. Characteristics of healthy donors and AS patients**

|  | Healthy donors | Patients with AS |
| --- | --- | --- |
| Number | 12 | 10 |
| Age (years) | 21.6± 8.0 | 22.8 ± 8.2 |
| Males, no. (%) | 11 (91.7%) | 9 (90.0%) |
| HLA-B27-positive, no. (%) | 0 | 8 (80.0%)* |
| Disease duration (years) | NA | 3.9 ± 3.5 |
| CRP (mg/L) | 3.7 ± 2.1 | 26.9 ± 16.4* |
| ESR (mm/h) | 14.6 ± 10.9 | 63.5 ± 28.3* |
| BASDAI | 1.1 ± 0.8 | 5.3 ± 1.1* |

Means ± SD. AS, ankylosing spondylitis; HLA-B27, human leukocyte antigen B27; CRP,

C-reactive protein; ESR, erythrocyte sedimentation rate; BASDAI, bath ankylosing

spondylitis disease activity index; * P< 0.05 compared to healthy donors.
