# Supplementary figures and images for: Aberrant lncRNA–mRNA expression profile and function networks during the adipogenesis of mesenchymal stem cells from patients with ankylosing spondylitis
Source: Front Genet. 2022 Oct 3;13:991875. doi: 10.3389/fgene.2022.991875 (PMC9563993; doi:10.3389/fgene.2022.991875)

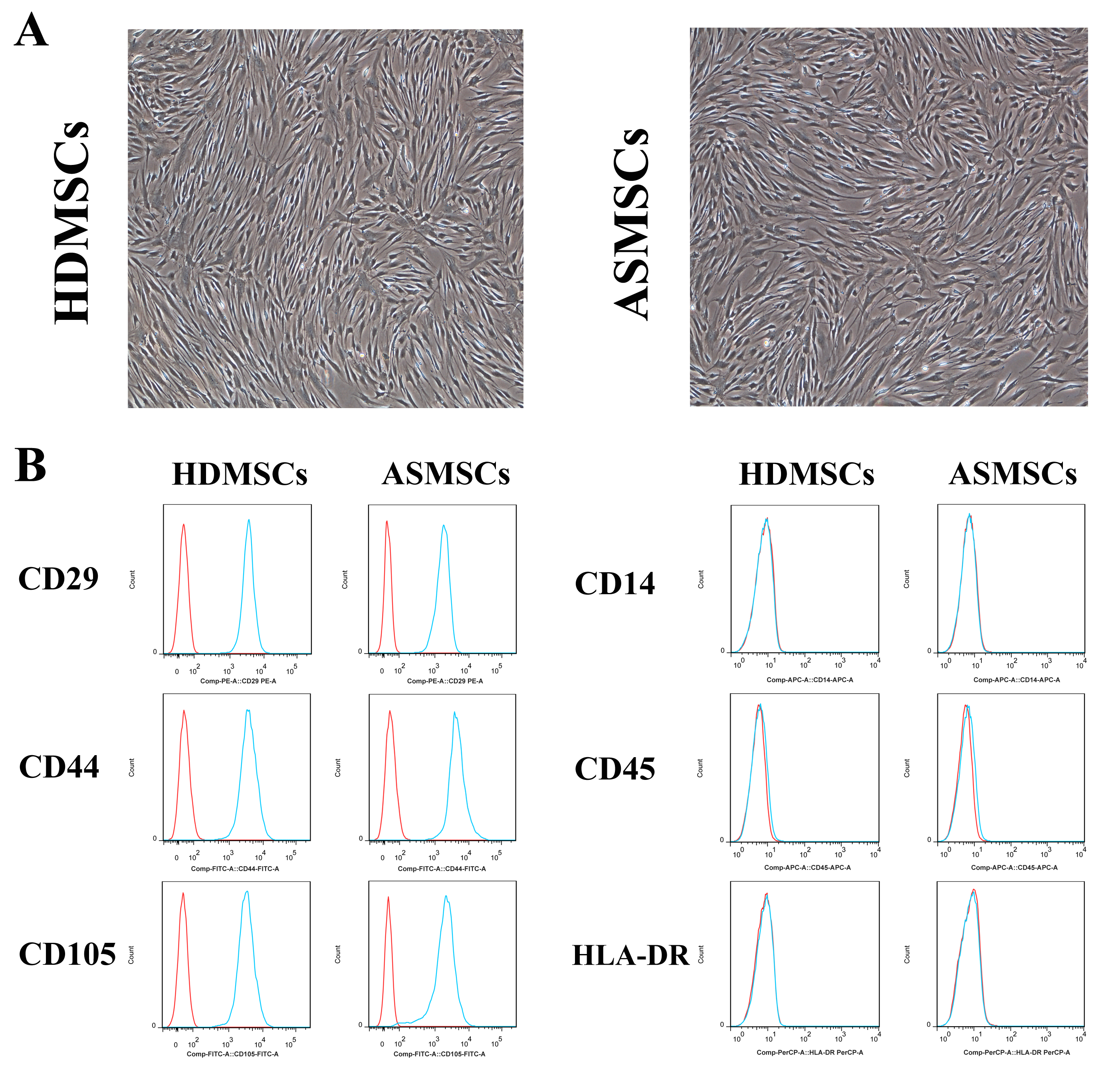

Supplement: Supplementary file 2 [file Image1.TIF]
